# Impact of urban green space on self-rated health: Evidence from Beijing

**DOI:** 10.3389/fpubh.2022.999970

**Published:** 2022-09-08

**Authors:** Dongsheng Zhan, Qianyun Zhang, Mei-Po Kwan, Jian Liu, Bochuan Zhan, Wenzhong Zhang

**Affiliations:** ^1^School of Management, Zhejiang University of Technology, Hangzhou, China; ^2^Department of Geography and Resource Management, Institute of Space and Earth Information Science, The Chinese University of Hong Kong, Shatin, Hong Kong SAR, China; ^3^College of Resource Environment and Tourism, Capital Normal University, Beijing, China; ^4^School of Health Services Management, Anhui Medical University, Hefei, China; ^5^Key Laboratory of Regional Sustainable Development Modeling, Institute of Geographic Sciences and Natural Resources Research, Chinese Academy of Sciences, Beijing, China

**Keywords:** urban green space, self-rated health, influencing mechanism, binary logistic model, Beijing

## Abstract

As a crucial component of urban green space (UGS), urban parks have been found to be closely associated with the health of urban residents. Drawing on a large-scale survey, “International First-class Harmonious and Livable Capital”, in Beijing conducted in 2018, this paper examines the impact of subjective and objective characteristics of UGS on residents' self-rated health (SRH) by using a binary logistic regression model. The results indicate that the overall SRH status of urban residents in Beijing is relatively good, with 73.8% of the respondents reporting good SRH. The perceived quality of UGS and objectively measured accessibility to UGS are positively associated with residents' SRH, but the subjective indicator of UGS has a greater impact on SRH than the objective indicator of UGS. In terms of influencing mechanisms, social interaction and air quality perception were the two major mediators of UGS that affected residents' SRH. The heterogeneity analysis suggests that objective accessibility to different types of urban parks has mixed effects on residents' SRH. Access to high-quality parks is positively associated with residents' SRH, whereas access to common parks has a negative impact on residents' SRH. Our findings provide important policy implications for optimizing urban park design and improving the quality of urban park provision according to human needs in the Beijing Metropolitan Area.

## Introduction

Urbanization is advancing rapidly worldwide, with more than 60% of the global population living in cities. However, rapid, extensive urbanization in some developing countries such as China has been accompanied by serious environmental pollution and a high incidence of chronic diseases (1, 2). Since Healthy China 2030 was proposed in 2016 by the Chinese government, public health concerns have received widespread attention from interdisciplinary scholars (3). Most health studies have focused on either objective health (i.e. mortality and morbidity due to various diseases) or subjective health (i.e. self-rated health [SRH]) (4, 5). More recently, a growing body of health studies has considered SRH a useful measurement indicator of public health because of data availability at a fine spatial scale and its satisfactory prediction of mortality (6).

The factors that influence individuals' health in the research community comprise both intrinsic and extrinsic factors. Intrinsic factors are some physiological factors and behaviors, for example, daily health behaviors and genetic factors that are potential determinants affecting individuals' health. For instance, health behaviors such as individual dietary habits and physical activity may directly affect human health (7). Health tends to vary according to individuals' behavior and socioeconomic status (8). External factors such as the natural environment, social capital, level of economic development, and the built environment are also associated with health and can be broadly classified into three levels. At the macro level, factors such as urbanization level, socioeconomic development level, and availability of medical facilities exert different degrees of impact on residents' health (9). To build a healthy city, the residential environment is also of substantial concern in recent health-related research at the meso level (10). Evidence has suggested that neighborhood social capital, community cohesion, and perceived neighborhood deprivation play important roles in determining the physical and psychological health of residents (11, 12). Other scholars have focused on health at the micro level and analyzed the effect of environmental exposure on residents' health through individual spatiotemporal behaviors, such as residents' travel mode and commuting time (13). However, most health-related studies at the micro level have been conducted in developed countries, such as the United States and the United Kingdom (14, 15), and there has been scant research on the association between individuals' residential environments and their health in China.

Urban green space (UGS) is green infrastructure, namely, for example, urban parks, urban forests, public green spaces, school playgrounds, public rest areas, city squares, and vacant lots (16), among which urban parks are one of the most critical components and are widely used by nearby residents. In recent years, urban construction land has expanded rapidly due to rapid urbanization, especially in developing countries (17), encroaching on natural resources and ecological land within the city limits. The continuous reduction of UGS, has increased the incidence of various mental illnesses and chronic diseases (18, 19). With the increasing human demand and numerous health benefits of green spaces, UGS has also become an emerging research focus in health studies. However, most of the urban greenery and health literature has been conducted in the western context on relatively low-density cities, and their findings cannot be generalized to Chinese cities that have a higher population density.

UGS provides a wide variety of social, economic, and environmental benefits (20, 21). Existing health-related studies have found that UGS is positively associated with residents' health through various mechanisms in the physical, psychological, and social dimensions (22). The typical theories linking UGS and health are stress reduction theory and attention restoration theory (23, 24), and the health effects of UGS are mainly composed of three mediated paths (21). The first mediated path is the reduction of environmental stress, which means that UGS can effectively mitigate the harmful impact of air pollution, noise pollution, and other types of deleterious environmental exposures in the living environment (25). The second mediated path is restoring capacity, which shows that UGS can relieve residents' physical and psychological stress and restore their attention, reducing the prevalence of chronic diseases (26). The third mediated path is building capacity, through which UGS can improve the living environment for physical exercise and thus enhance residents' physical fitness (27). Additionally, UGS, considered a high-quality social activity space, can also promote social interaction, which enhances residents' social well-being and mental health (28). Although burgeoning literature has focused on the relationship between UGS and residents' health, few studies have examined the impact of both objectively measured access to UGS and the perceived quality of UGS on residents' SRH.

Research has provided evidence of the UGS-health association. However, most of the health-related literature has measured UGS only from an objective perspective, such as the quantity and accessibility of UGS (29). Widely used accessibility measurement methods are, for example, the buffer zone method, shortest distance method, Gaussian two-step floating catchment area method, and gravity model method (30, 31). However, evidence showed that proximity to UGS has a mixed effect on residents' health. For instance, some studies have found that the increasing quantity of UGS near the residence tends to relieve residents' psychological stress and thus promote their mental health (32); other studies have found a negative association between UGS and residents' health after controlling for their individual characteristics (33). This is in part due to the fact that there are limitations to only consider the quantity or accessability of the UGS while neglecting their quality. Numerous previous studies have reported the varied health enhancing effects of the UGS among diffent types of urban parks (e.g., size and quality) (34, 35). Another possible reason for the inconsistent results may be induced by spatial mismatch between the distribution of UGS and local residents' real needs (36), because utilizing only objectively measured accessibility to UGS may ignore the actual needs of residents related to UGS. Therefore, examining residents' perceived quality of UGS from an environmental psychological perspective is necessary.

With the popularity of individual-centered urban development, the relationship between individuals' perceptions of UGS and their health has received increasing attention, and the concept of perceived accessibility has been widely used. Perceived accessibility generally refers to an individual's subjective perception of and satisfaction with the accessibility to public facilities in the physical environment (37), indicating a comprehensive understanding of objective accessibility, quality, and other use processes of the UGS. Although both objective and subjective characteristics of UGS could potentially affect residents' actual use behavior of UGS and further affect their health (38), few studies have focused on the impact of both subjective and objective measurement indicators of UGS on residents' health.

In filling these research gaps, exploring the association between UGS and residents' SRH using both subjective and objective perspectives is necessary. As an influential component of UGS, urban parks have a close relationship with residents' daily lives and health. Thus, exploring the impact and influence mechanisms of urban parks on residents' health to promote the construction of UGS as well as sustainable and healthy urban development is important. Drawing on a large-scale questionnaire survey, “International First-class Harmonious and Livable Capital”, conducted in Beijing in 2018, this study combined both objective accessibility indicators and subjective perceptions of UGS to examine the relationship between UGS and urban residents' SRH in Beijing using a binary logistic regression model while controlling for residents' socioeconomic attributes.

More specifically, the objectives of this study are to (1) compare the impact intensity of both objectively and subjectively measured indicators of UGS in influencing residents' SRH; (2) explore the mediating paths through which UGS affects the SRH of urban residents; and (3) examine the heterogeneous effects of different types of urban parks on residents' SRH. Our findings provide policy insights into optimizing the allocation of UGS and improving human health through improved UGS provision.

## Materials and methods

### Research framework

**Figure 1:**
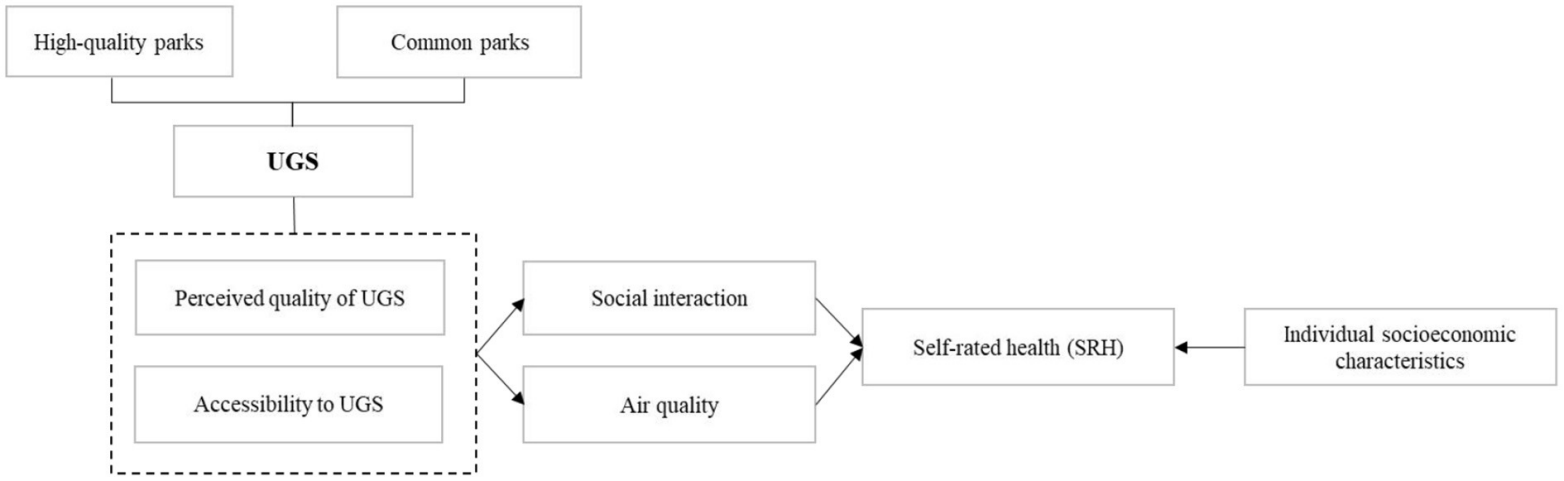
Research framework for the association between UGS and residents' SRH.

As shown in Figure 1, this study takes Beijing as a case study and constructs a research framework for examining the impact of UGS on residents' SRH from both objective and subjective perspectives, in which subjective perceptions of UGS reflect respondents' satisfaction with UGS quality and objective UGS accessibility (distance from the respondents' residence to the nearest urban park). By referring to the literature (21) and considering data availability, this study focuses primarily on the mediating mechanisms of both social interaction and air quality for UGS in influencing residents' SRH, and socioeconomic characteristics are also included as control variables in the analytical framework (34).Since high-quality parks generally provide healthier and more enjoyable environmental elements than common parks, thus the heterogenous effects of different types of urban parks (e.g., high-quality parks and common parks) on residents' SRH is also considered in our study.

### Study area and data sources

**Figure 2:**
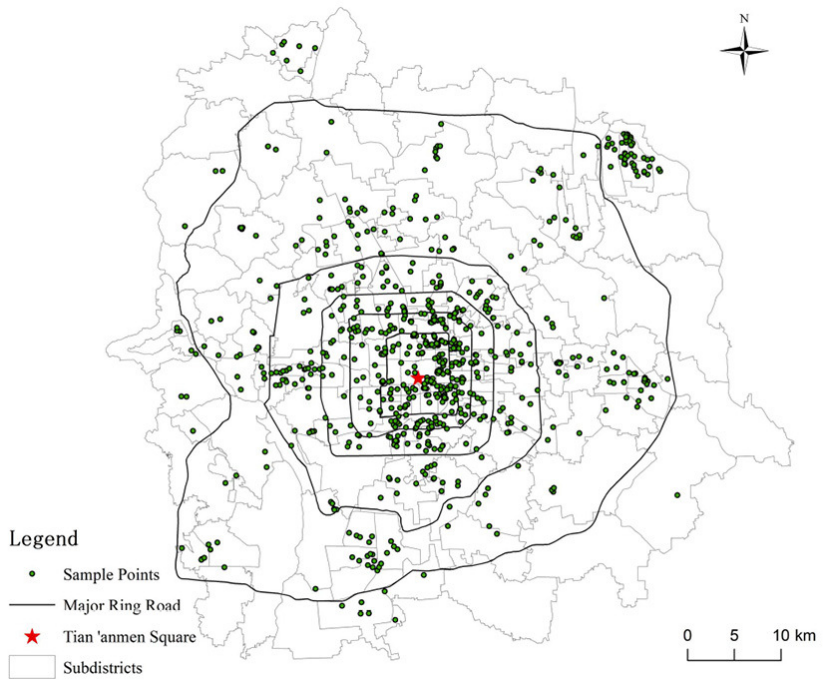
Spatial distribution of study area and surveyed respondents in Beijing.

The questionnaire survey “International First-class Harmonious and Livable Capital” was conducted in the urban areas in Beijing in April 2018. It was performed by the Institute of Geographic Sciences and Natural Resource Research of the Chinese Academy of Sciences and covered 184 streets within the sixth ring road. Surveyed respondents were randomly selected from the streets with support from the local community council, and a face-to-face questionnaire survey was conducted with trained investigators. Because our survey covered many streets, it is representative of all types of urban residents' perceived living environment quality and SRH in Beijing. The spatial distribution of the study area and the surveyed respondents are shown in Figure 2.

The survey covered seven dimensions of living environment quality (urban safety, life convenience, comfort of the natural environment, comfort of the human environment, convenient transport, openness and innovation, and urban management) and respondents' socioeconomic characteristics such as age, gender, marital status, educational attainment, annual household income, car ownership, household registration, and occupation type. The survey also included residents' SRH and perceived quality of the park near the residential area. At the same time, in order to explore the mediating role of social interaction and air quality in the process of park affecting residents' health, we also investigated respondents' satisfaction with these two items.Our survey also collected the geographic coordinates of respondents' residences, with the support of a location-based service, which can be applied to accurately identify respondents' accessibility to UGS. The total number of questionnaires obtained in our survey was 10,651. After removing questionnaires with missing data, we finally have 10,011 effective surveys, with an effective rate of 93.99%.

### Variables

#### Self-rated health

SRH is a widely used indicator of public health because of its easy availability in large-scale surveys (39) and its importance in predicting mortality (40). Hence, SRH was measured using a five-point Likert scale to assess the overall perceived physical and psychological health of the respondents in this study. All respondents were asked, “How do you feel about your health compared with your peers?” The response items comprised five options related to residents' health: “very good”, “good”, “fair”, “bad”, and “very bad”. To avoid the potential bias caused by sparse data (41), the original five-point Likert scale data were dichotomised into good SRH (i.e. “very good” and “good”) and poor SRH (i.e. “fair”, “bad”, “very bad”). In our model, good SRH was coded as 1, and poor SRH was coded as 0.

#### Indicators of urban green space

In this study, UGS indicators comprised objective and subjective aspects (42). Because urban parks are a typical UGS closely related to residents' daily lives, this study selected urban parks as a proxy variable for UGS and examined 384 registered parks in the urban areas of Beijing. The objective indicator of UGS, focusing on its accessibility, was defined as the shortest distance to the UGS, measuring the spatial distance obstacle from a person's residence to the nearest park. Notably, objectively measured accessibility was an inverse index, where a shorter distance indicated higher accessibility. Because urban parks are daily recreational spaces for residents and essential green infrastructure in the city, most urban parks are located within 1 km of residential areas, leading to a low travel resistance for urban residents to nearby urban parks. The minimum distance method is suitable for measuring accessibility to a UGS (Nielsen and Hansen, 2007). For the subjective indicator of UGS, we used a special question in the questionnaire to measure participants perceived quality of UGSs. The question uses a five-point Likert scale, and the respondents were asked, “How satisfied are you with your surrounding parks and UGS?” The response items comprised “very dissatisfied”, “dissatisfied”, “average”, “satisfied” and “very satisfied”, and their values were 1 to 5 points, respectively.

#### Mediator variables

In this study, social interaction and air quality were used as mediator variables, both of which were measured through respondents' perceived evaluations of living environment quality. In terms of social interaction, respondents were asked, “How satisfied are you with your social interactions in the community?” As for air quality, respondents were asked, “How satisfied are you with the air quality around your residential area?” For both survey questions, response items such as “strongly dissatisfied”, “dissatisfied”, “fair”, “satisfied”, and “very satisfied” were assigned a score from 1 to 5, respectively.

#### Socioeconomic characteristics

The control variables in this study were the socioeconomic characteristics of the respondents, such as age, gender, marital status, educational attainment, annual household income, car ownership, household registration, and occupation type. The distance to the city center from the respondents' residence was also controlled, which can reflect residents' residential location. Table 1 lists descriptive statistics of all variables.

**Table 1:** Descriptive statistics of all variables.

**Variables**	**Definition**	**Mean**	**S.D**.	**Min**.	**Max**.
SRH	Self-rated health	3.928	0.738	1	5
Perceived quality of UGS	Respondents' subjective perception of UGS quality	3.982	0.703	1	5
Accessibility to UGS	Distance to the nearest urban park from the respondents' residence	1.373	1.084	0.024	8.656
Accessibility to high-quality park	Distance from the respondents' residence to the nearest urban high-quality park	2.778	2.612	0.066	15.16
Accessibility to common park	Distance from the respondents' residence to the nearest urban common park	1.817	1.235	0.024	8.656
**Mediating variables**					
Social interaction	Satisfaction with social interactions in the community	3.849	0.680	1	5
Air quality	Satisfaction with air quality around the residential area	3.342	0.904	1	5
**Socioeconomic characteristics**s					
DisCBD	Distance to city center	15.210	8.150	0.694	40.36
Male	Dummy: 1 = Male, 0 = else	0.480	0.500	0	1
Female	Dummy: 1 = Female, 0 = else	0.520	0.500	0	1
Age 1	Dummy: 1 = Under 20 years old, 0 = else	0.040	0.197	0	1
Age 2	Dummy: 1 = 20–29 years old, 0 = else	0.189	0.391	0	1
Age 3	Dummy: 1 = 30–39 years old, 0 = else	0.237	0.425	0	1
Age 4	Dummy: 1 = 40–49 years old, 0 = else	0.182	0.386	0	1
Age 5	Dummy: 1 = 50–59 years old, 0 = else	0.169	0.374	0	1
Age 6	Dummy: 1 = 60–69 years old, 0 = else	0.143	0.350	0	1
Age 7	Dummy: 1 = 70 years and above, 0 = else	0.040	0.195	0	1
Married	Dummy: 1 = Married, 0 = else	0.765	0.424	0	1
Unmarried	Dummy: 1 = Unmarried, 0 = else	0.235	0.424	0	1
Edu 1	Dummy: 1 = Middle school and below, 0 = else	0.165	0.371	0	1
Edu 2	Dummy: 1 = High School, 0 = else	0.299	0.458	0	1
Edu 3	Dummy: 1 = College, 0 = else	0.284	0.451	0	1
Edu 4	Dummy: 1 = University, 0 = else	0.201	0.401	0	1
Edu 5	Dummy: 1 = Graduate student and above, 0 = else	0.051	0.220	0	1
Occupation 1	Dummy: 1 = State-owned enterprises, 0 = else	0.156	0.363	0	1
Occupation 2	Dummy: 1 = Non-state-owned enterprises, 0 = else	0.844	0.363	0	1
Income 1	Dummy: 1 = Below 30,000 CNY, 0 = else	0.055	0.228	0	1
Income 2	Dummy: 1 = 30,000–50,000 CNY, 0 = else	0.087	0.282	0	1
Income 3	Dummy: 1 = 50,000–100,000 CNY, 0 = else	0.188	0.390	0	1
Income 4	Dummy: 1 = 100,000–200,000 CNY, 0 = else	0.502	0.500	0	1
Income 5	Dummy: 1 = 200,000–300,000 CNY, 0 = else	0.111	0.315	0	1
Income 6	Dummy: 1 = 300,000–500,000 CNY, 0 = else	0.039	0.195	0	1
Income 7	Dummy: 1 = 500,000–1,000,000 CNY, 0 = else	0.015	0.120	0	1
Income 8	Dummy: 1 = Above 1,000,000 CNY, 0 = else	0.002	0.045	0	1
Car 1	Dummy: 1 = Owning at least one car, 0 = else	0.531	0.499	0	1
Car 2	Dummy: 1 = Owning no car, 0 = else	0.469	0.499	0	1
Hukou 1	Dummy: 1 = Beijing Hukou, 0 = else	0.681	0.466	0	1
Hukou 2	Dummy: 1 = Non-Beijing Hukou, 0 = else	0.319	0.466	0	1

### Research method

In this study, a binary logistic regression model was used to examine the effects of UGS on urban residents' SRH in Beijing. First, the subjective indicator (respondents' perceived UGS quality) was included in the model as the key explanatory variable (Model 1a). Second, the objective indicator of UGS, measured by accessibility (the distance to the nearest urban park from the respondent's residence), was included as the key explanatory variable in the model (Model 1b). Finally, both accessibility to UGS and residents' perceptions of UGS were included in the model (Model 1c), to test the joint effect of the subjective and objective measures of UGS on individuals' SRH.

The control variables were the distance to the city center and repondents' socioeconomic characteristics, such as gender, age, marital status, educational attainment, occupation type, total annual household income, car ownership, and *hukou*. The logistic regression model equation is as follows:


$$\begin{array}{l} \text{Logit}\;\text{P}\;=\text{Ln}(\frac{\text{P}}{1-\text{P}})\;=\;\text{α}+{\text{β}}_{\text{i}}{\text{x}}_{\text{i}}+{\text{β}}_{\text{j}}{\text{x}}_{\text{j}} \end{array}$$

where P denotes Y = 1, the probability of occurrence of respondents having good SRH; 1-P denotes Y = 0, the probability of occurrence of respondents with poor SRH; α is the intercept; *x*_*i*_ and β_*i*_ are UGS variables and the corresponding regression coefficients; *x*_*j*_ and β_*j*_ are control variables and the corresponding regression coefficients.

## Results

### Socioeconomic characteristics and self-rated health outcome of respondents

Table 2 summarizes respondents' demographic and socioeconomic characteristics. The respondents were mainly young and middle-aged (20–49 years old): these two groups accounted for approximately 60% of all respondents. The gender of the respondents showed little difference: male and female respondents accounted for 47.9 and 52.1%, respectively. The percentage of married and unmarried respondents was 76.5 and 23.5%, respectively. In terms of educational attainment, 53.6% had a college education or above, indicating high educational attainment among the respondents. Regarding occupation type, 15.6% of respondents worked in state-owned enterprises. Additionally, 66.9% of the respondents reported an annual household income of 100,000 CNY or above, and 53.1% of the respondents had private cars. Residents of Beijing with hukou accounted for 68.1% of the respondents, and 31.9% of the respondents were migrants. Overall, the demographic and socioeconomic characteristics of the respondents in our investigation are similar to the characteristics of the general population in Beijing.

**Table 2:** Demographic and socioeconomic characteristics of respondents.

**Factors**	**Percentage (%)**	**Factors**	**Percentage (%)**
**Gender**		**Occupation type**	
Male	47.9	State-owned enterprises	15.6
Female	52.1	Non-state-owned enterprises	84.4
**Age**		**Household income**	
<20 years old	4.0	<30,000 CNY	5.5
20–29 years old	18.9	30,000–50,000 CNY	8.7
30–39 years old	23.7	50,000–100,000 CNY	18.8
40–49 years old	18.2	100,000–200,000 CNY	50.2
50–59 years old	16.9	200,000–300,000 CNY	11.1
60–69 years old	14.3	300,000–500,000 CNY	3.9
≥70 years old	4.0	500,000–1,000,000 CNY	1.5
**Marriage status**		>1,000,000 CNY	0.2
Married	76.5	**Car ownership**	
Unmarried	23.5	Yes	53.1
**Education**		No	46.9
Middle school and below	16.5	**Household registration**	
High school	29.9	Beijing *Hukou*	68.1
College	28.4	Non-Beijing *Hukou*	31.9
University	20.1		
Graduate student and above	5.1		

**Figure 3:**
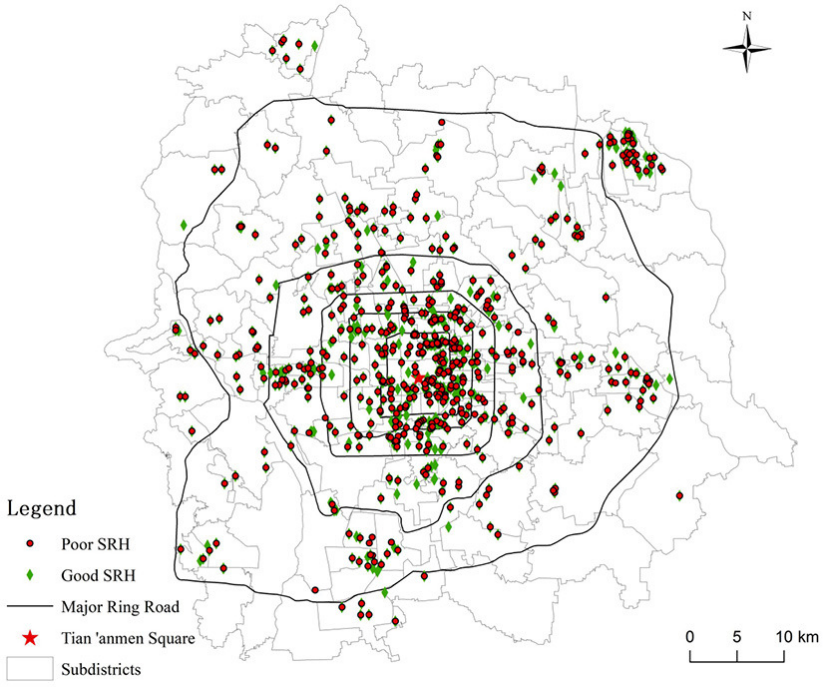
Spatial distribution of SRH among respondents.

Figure 3 shows the spatial distribution of the respondents' SRH outcomes. According to the descriptive statistics of participants' SRH, the mean score of their SRH is 3.93 with a standard deviation of 0.703, indicating that urban residents in Beijing generally have good SRH. After the original SRH variable was dichotomised, 73.8% of respondents had good SRH and 26.2% had poor SRH.

### Objective and subjective characteristic of urban green space attributes

Table 3 shows the descriptive statistics of participants' perceived quality of and accessibility to UGS. Their average distances to the nearest UGS, such as all urban parks, high-quality parks, and common parks, are 1.37, 2.78, and 1.82 km, respectively. The mean score of respondents' perceived quality of the UGS was 3.98, suggesting that they were generally satisfied with the UGS in Beijing.

**Table 3:** Descriptive statistic of perceived quality and accessibility to UGS.

**Variables**	**Mean**	**St. Dev**.
Perceived quality of UGS	3.98 points	0.703
Accessibility to UGS	1.37 km	1.084
Accessibility to high-quality park	2.78 km	2.612
Accessibility to common park	1.82 km	1.235

### Impacts of urban green space on residents' self-rated health

A binary logistic regression model was used to examine the effect of UGS on participants' SRH. In order to compare the effects of influencing factors, all explanatory variables were standardized before being introduced into the model. The results of the multicollinearity test showed that the variance inflation factor (VIF) values of all independent variables were below 5, indicating that multicollinearity is not a problem. The results of the regression model are shown in Table 4. Model 1a shows the relationship between the perceived quality of UGS and respondents' SRH from a subjective perspective. The odds of participants reporting good SRH is positively associated with their perceived quality of UGS, with regression coefficients and odds ratios of 0.329 and 1.390, indicating that a one-unit increase in participants' satisfaction with the surrounding parks and UGSs increases the odds of reporting good SRH by 39.0%. Model 1b shows the relationship between accessibility to UGS and participants' SRH from an objective perspective. The odds of respondents reporting good SRH are positively associated with accessibility to UGS, with coefficients and odds ratios of 0.049 and 1.050, respectively, indicating that the odds of participants reporting good SRH increase by 5.0% for each unit increase in distance to the nearest urban park. Model 1c shows the effects of the perceived quality of UGS and accessibility of UGS on SRH from a combined subjective and objective perspective. Despite the smaller regression coefficient in Model 1c, both perceived quality of UGS and accessibility to UGS exerted significant and positive effects on the odds of reporting good SRH among respondents, with odds ratios of 1.389 and 1.048, respectively. By contrast, the impact intensity of perceived quality of UGS on participants' SRH is much greater than that of accessibility to UGS.

**Table 4:** Regression model results of the association between UGS and respondents' SRH.

	**Model 1a**			**Model 1b**			**Model 1c**		
**Variables**	**Coefficient**	**OR**	**S.E**.	**Coefficient**	**OR**	**S.E**.	**Coefficient**	**OR**	**S.E**.
Perceived quality of UGS	0.329^***^	1.390	0.046				0.329^***^	1.389	0.046
Accessibility to UGS				0.049^**^	1.050	0.024	0.047^**^	1.048	0.024
DisCBD	0.036	1.036	0.038	0.013	1.013	0.037	0.028	1.029	0.038
Male	0.075	1.078	0.051	0.081^*^	1.084	0.051	0.074	1.077	0.051
Age 1	Reference			Reference			Reference		
Age 2	−0.463^***^	0.629	0.104	−0.479^***^	0.619	0.102	−0.464^***^	0.629	0.104
Age 3	−0.467^***^	0.627	0.108	−0.502^***^	0.606	0.104	−0.467^***^	0.627	0.108
Age 4	−0.694^***^	0.499	0.086	−0.714^***^	0.490	0.084	−0.692^***^	0.500	0.087
Age 5	−0.789^***^	0.454	0.079	−0.832^***^	0.435	0.075	−0.791^***^	0.453	0.079
Age 6	−1.313^***^	0.269	0.047	−1.363^***^	0.256	0.044	−1.308^***^	0.270	0.047
Age 7	−1.406^***^	0.245	0.047	−1.511^***^	0.221	0.042	−1.400^***^	0.247	0.048
Married	−0.020	0.981	0.074	−0.028	0.972	0.073	−0.023	0.978	0.074
Edu 2	−0.028	0.973	0.070	−0.014	0.986	0.070	−0.024	0.976	0.070
Edu 3	0.101	1.106	0.091	0.113	1.120	0.091	0.102	1.108	0.092
Edu 4	−0.049	0.952	0.093	−0.032	0.969	0.094	−0.045	0.956	0.093
Edu 5	−0.383^***^	0.682	0.098	−0.410^***^	0.664	0.095	−0.382^***^	0.683	0.098
Occupation type 2	−0.082	0.921	0.063	−0.055	0.947	0.065	−0.076	0.927	0.064
Income 2	0.482^***^	1.620	0.192	0.461^***^	1.585	0.186	0.484^***^	1.622	0.192
Income 3	0.724^***^	2.062	0.220	0.710^***^	2.033	0.215	0.725^***^	2.064	0.220
Income 4	0.718^***^	2.051	0.235	0.714^***^	2.042	0.232	0.723^***^	2.060	0.236
Income 5	0.945^***^	2.574	0.354	0.948^***^	2.581	0.354	0.954^***^	2.596	0.357
Income 6	1.042^***^	2.835	0.499	1.019^***^	2.770	0.485	1.058^***^	2.879	0.508
Income 7	0.448^*^	1.565	0.353	0.459^*^	1.582	0.355	0.457^*^	1.579	0.357
Income 8	−0.001	0.999	0.485	−0.085	0.919	0.441	0.013	1.013	0.492
Car 1	−0.079	0.924	0.068	−0.083	0.920	0.067	−0.085	0.918	0.067
hukou 1	−0.377^***^	0.686	0.038	−0.378^***^	0.685	0.038	−0.377^***^	0.686	0.038

*** p < 0.01

** p < 0.05,

* p < 0.1; reference groups are Age 1, unmarried, edu 1, Occupation type 1, income 1, car 2, and hukou 2.

In addition, the results of Model 1c show that some socioeconomic attributes are also associated with respondents' SRH. Middle-aged and elderly residents had lower odds of reporting good SRH than residents aged under 20 years. Respondents with a graduate education or above had a poorer SRH than residents with a below middle school education, with a 31.7% lower odds ratio reporting good SRH. Residents with a higher annual household income had better SRH than the reference group with annual household income below 30,000 CNY, with the odds ratio ranging from 1.565 in the 300,000–500,000 CNY group to 2.835 in the 500,000–1,000,000 CNY group. Regarding household registration, respondents with a Beijing hukou had worse SRH than non-hukou residents, with a 31.4% decrease in the odds of residents reporting good SRH.

To explore the specific influencing mechanisms of the effects of UGS on respondents' SRH, social interaction and air quality were added as mediating variables in the model. The results are shown in Table 5. Models 2a and 2b examine the effects of the subjective and objective indicators of UGS on the mediating variables of social interaction and air quality, which are also new dependent variables. The modeling results showed that respondents' perceived quality of UGS was significantly and positively associated with social interaction and air quality, with regression coefficients of 0.433 and 0.561, respectively. However, accessibility to UGS was positively associated only with air quality, with a regression coefficient of 0.203.

**Table 5:** Mediating effect of the association between UGS and respondents' SRH.

	**Model 2a**			**Model 2b**			**Model 2c**		
	**Social interaction**			**Air quality**			**SRH**		
	**Coefficient**	**OR**	**Std. Err**.	**Coefficient**	**OR**	**Std. Err**.	**Coefficient**	**OR**	**Std. Err**.
Social interaction							0.060^*^	1.062	0.036
Air quality							0.196^***^	1.216	0.028
Perceived quality of UGS	0.433^***^	1.542	0.085	0.561^***^	1.753	0.167	0.269^***^	1.309	0.036
Accessibility to UGS	0.026	1.026	0.075	0.203^*^	1.225	0.114	0.044	1.044	0.022
Covariates	controlled			controlled			controlled		

*** p < 0.01,

^**^p < 0.05,

* p < 0.1.

Model 2c included two mediating variables, social interaction and air quality, in the regression analysis based on the baseline model. The results showed that social interaction and air quality were significantly and positively associated with residents' SRH, with regression coefficients of 0.060 and 0.196, respectively. The mediating analysis results reveal that social interaction and air quality are important mediator variables for the UGS effect on respondents' SRH, and UGS affects respondents' SRH directly or indirectly by increasing their satisfaction with social interaction and air quality.

### Heterogeneity effects of types of urban green space

Following the high-quality parks rating standard issued in Beijing, local authorities have differentiated urban parks into high-quality parks and common parks based on a set of criteria since 2002, such as planning and construction, greening and maintenance, supporting facilities, and order maintenance. Specifically, high-quality parks must fulfill the following criteria: (1) a park green space cover rate of over 70%; (2) a satisfaction ratio among the visitors that exceeds 90%; (3) the loess in the park is not open to air; (4) neat, standardized signage; two-star or higher toilets; (5) no advertising umbrellas or other facilities that hinder the landscape; (6) no stagnant water, dirt, spitting, or cigarette butts; no vending stalls within 50 m of the park entrance; (7) good order in the park; (8) no major complaints or safety accidents. High-quality parks generally provide healthier and more liveable environmental elements than common parks.

To examine the heterogeneous effects of types of urban parks on respondents' SRH, we differentiated urban parks into high-quality and common parks for further analysis. Table 6 shows the model results for high-quality parks (Model 3a) and common parks (Model 3b). The results of Model 3a show that the accessibility to UGS for high-quality parks is significantly and positively associated with respondents' SRH, with a regression coefficient of 0.085, indicating that the odds of reporting good SRH increase by 8.9% when the distance to a high-quality park increases by one unit for residents. The results of Model 3b show that accessibility to UGS for common parks is negatively associated with respondents' SRH, with a regression coefficient of −0.057, indicating that the odds of residents reporting good SRH decreases by 5.6% when the distance to common parks increases by one unit.

**Table 6:** Heterogeneous effects of urban park types on respondents' SRH.

	**Model 3a Park type** = **high-quality parks**			**Model 3b Park type** = **common parks**		
	**Dependent variable: SRH**			**Dependent variable: SRH**		
	**Coefficient**	**OR**	**Std. Err**.	**Coefficient**	**OR**	**Std. Err**.
Perceived quality of UGS	0.377^***^	1.458	0.046	0.375^***^	1.455	0.046
Accessibility to UGS	0.085^***^	1.089	0.028	−0.057^*^	0.944	0.028
Covariates	controlled			controlled		

*** p < 0.01,

^**^p < 0.05,

* p < 0.1.

## Discussion

### Main findings and contributions to existing work

This study explored the relationship between UGS and respondents' SRH using data collected in a large-scale survey in Beijing: the International First-class Harmonious and Liveable Capital. Our study contributes to the literature on UGS and health in at least three aspects. First, we combine the objective and subjective characteristics of UGS in the research framework. Second, mediating mechanisms such as social interaction and air quality between the UGS and residents' health are examined. Finally, our study adds new empirical evidence on the health impacts of UGS in developing countries with rapid urbanization, using Beijing as a case study.

Our study found that the mean value of respondents' SRH in Beijing was 3.93 and 73.8% of residents reported good SRH, indicating that the overall SRH of urban residents in Beijing was good. This finding is similar to that in many studies (43): Beijing, the capital of China, has many beneficial conditions such as a high level of economic development, satisfactory medical facilities, and conveniently access to UGS. In addition, urban residents in Beijing, characterized by good education in general, have a healthy lifestyle and good health knowledge, which contribute to their reporting a good SRH.

The binary logistic regression model results indicated that the perceived quality of UGS was positively associated with respondents' SRH. This finding occurs because residents' perceived quality or satisfaction with urban parks originates from their experience (37), and residents' physiological health can be improved by using UGS (44), enhancing their SRH. Unexpectedly, accessibility to UGS was positively associated with residents' SRH in this study, indicating that residents with a longer distance from the nearest urban park have better SRH than those with a shorter distance, which was different from the results in the literature (45). A possible reason is that park facilities will crowd out the spatial layout of other public service facilities. Thus, the park layout should consider avoiding crowding other public service facilities. Different residents have different perceptions of and satisfaction with the same environment, and objective UGS accessibility indicators cannot fully reflect residents' subjective perceptions of UGS and their health benefits. Therefore, in the design of park UGS, the perception factors of residents should be fully considered to improve the role of park UGS in promoting health.

In line with the literature (46, 47), our findings showed that social interaction and air quality are important pathways through which UGS affects respondents' SRH. Specifically, the perceived quality of UGS positively contributes to respondents' SRH by enhancing their satisfaction with both social interaction and air quality, and accessibility to UGS was only relevant for respondents' SRH through satisfaction with air quality. UGS was shown to abate PM2.5 concentrations and effectively reduce the health risks caused by air pollution (48). In addition, UGS, as an area for residents' daily activities, creates a good public space for social interaction among residents (49). Increased social interaction is conducive to improving residents' social identity and alleviating negative emotions, such as anxiety (50), and promoting residents' physical and mental health.

The results of the heterogeneity analysis revealed that the effects of high-quality and common parks on respondents' SRH differed. Among them, accessibility to UGS for high-quality parks was positively correlated with respondents' SRH, indicating that respondents with a longer distance to the nearest high-quality park tend to report a good SRH. This result may be observed because high-quality parks generally occupy a large area, which, to a certain extent, compresses the space for other public service facilities, leading to other types of healthy living needs of residents not being fulfilled and, in turn, affecting respondents' SRH (51). By contrast, accessibility to UGS for common parks was negatively associated with respondents' SRH, suggesting that a longer distance to common parks for respondents tends to lead to poor SRH. These results conform to the results of Xie et al. (52), who also found that a shorter distance or better access to UGS is beneficial for the health of nearby residents. Thus, different optimisation measures should be implemented for high-quality parks and common parks to fulfill the diverse health needs of residents.

### Implications for park planning

The findings of our study provide important policy implications for improving UGS construction. Firstly, because the perceived accessibility of UGS plays a significant role in promoting residents' self-rated health, the planning and design of urban parks should pay attention to residents' perception of use and regularly collect residents' feedback on their satisfaction with urban parks. In addition, the objective distance of UGS also significantly affects the self-rated health of residents. Landscape planning departments should give priority to increasing urban parks with appropriate scale and high accessibility according to the distribution of residential areas and the urban road network system without crowding out other public service facilities.

Second, social interaction and air quality are important ways in which UGS affects SRH of respondents. Therefore, when designing urban parks, landscape design departments should not only provide good internal environment design and comfortable and pleasant green landscape to purify the air, but also create a comfortable atmosphere and provide convenient and diverse activity facilities to promote residents' social activities.

Finally, in view of the difference in the impact of high-quality parks and common parks on the SRH of respondents, the government departments in Beijing should take corresponding optimization measures for different types of parks. Specifically, the focus of optimisation for high-quality parks is to improve their quality, promote the transformation of high-quality parks into high-quality spaces, and fulfill the demands of some residents for high-end UGS. For common parks, the spatial quality and micro design of UGS in parks should be improved, based on not occupying land for other types of public service facilities. The area of park UGS should be appropriately expanded to increase fitness facilities and public spaces to fulfill the diversified needs of different groups with different social attribute.

### Limitations and future studies

This study has several limitations. First, this study used cross-sectional data, which cannot fully reveal the causal relationship and changing linkage between UGS and SRH among respondents. Second, the specific mediating mechanisms of UGS on respondents' SRH in our study only considered their perceived social interaction and air quality. Other potential mediating mechanisms (e.g., physical activity) were not explored and should also be included in further research. Finally, the accessibility to UGS in this study was measured only by the shortest road network distance without consideration of actual traffic speed in different grades of road segments and individuals' transport modes. Further research should focus on the impact of travel time accessibility to the UGS on residents' SRH. In addition, the areas of the original urban parks were aggregated into points according to their geometric gravity center, which could also cause some measurement bias in accessibility when encountering urban parks with a large area.

## Conclusions

Unlike developed western countries that have explored the health benefits of green space under the background of low population density, empirical evidence of green space and human health in developing countries with high population density is still relatively limited, and existing studies tend to focus only on the objective attributes of green space. Drawing on a large-scale survey and the spatial distribution of UGS in Beijing, this study quantified the association between UGS and residents' SRH by using a binary logistic regression model. It focused on the role of objectively measured accessibility to UGS in influencing residents' SRH, examined the relationship between perceived UGS quality and residents' SRH, and comprehensively measured the health benefits of UGS exposure levels from both the subjective and objective perspectives to mitigate the limitations of current research on the association between UGS and residents' health. In our study case, UGS is found to be associated with urban residents' SRH in Beijing. More specifically, both the perceived quality of UGS and objectively measured accessibility to UGS are positively related to residents' SRH, but the perceived quality of UGS has a much greater effect on residents' SRH. In addition, we identified that social interaction and air quality are important mediating paths through which UGS affects residents' SRH. Moreover, the effects of the types of urban parks on residents' SRH differed. Among these differences, accessibility to UGS for high-quality parks is positively associated with residents' SRH, and accessibility to UGS for common parks is negatively related to residents' SRH. Overall, this empirical evidence provides novel insights into optimizing green space,which could help guide planners and decision-makers to promote green space development for public health.

## Data availability statement

The original contributions presented in the study are included in the article/supplementary material, further inquiries can be directed to the corresponding authors.

## Author contributions

DZ, BZ, and WZ: conceptualization. DZ: writing and review. QZ: writing—original. M-PK: review and editing. JL: software. BZ and QZ: data analysis. WZ: investigation. All authors contributed to the article and approved the submitted version.

## Funding

This work was supported by the Humanities and Social Sciences Research Program of the Ministry of Education in China (20YJCZH221), National Natural Science Foundation of China (42001120), Fundamental Research Funds for the Provincial Universities of Zhejiang (GB202103004).

## Conflict of interest

The authors declare that the research was conducted in the absence of any commercial or financial relationships that could be construed as a potential conflict of interest.

## Publisher's note

All claims expressed in this article are solely those of the authors and do not necessarily represent those of their affiliated organizations, or those of the publisher, the editors and the reviewers. Any product that may be evaluated in this article, or claim that may be made by its manufacturer, is not guaranteed or endorsed by the publisher.

## References

[1] Sarkar C Webster C. Healthy cities of tomorrow: the case for large scale built environment–health studies. J Urban Health. (2017) 94:4–19. 10.1007/s11524-016-0122-1 28116584 PMC5359177

[2] Li X Song J Lin T Dixon J Zhang G Ye H. Urbanization and health in China, thinking at the national, local and individual levels. Environ Health. (2016) 15:113–23. 10.1186/s12940-016-0104-5 26961780 PMC4895783

[3] Tan X Liu X Shao H. Healthy China 2030: a vision for health care. Value Health Reg Issues. (2017) 12:112–4. 10.1016/j.vhri.2017.04.001 28648308

[4] Wang D Lau KK-L Yu R Wong SY Kwok TT Woo J. Neighbouring green space and mortality in community-dwelling elderly Hong Kong Chinese: a cohort study. BMJ Open. (2017) 7:e015794. 10.1136/bmjopen-2016-015794 28765127 PMC5642810

[5] Gascon M Triguero-Mas M Martínez D Dadvand P Forns J Plasència A. Mental health benefits of long-term exposure to residential green and blue spaces: a systematic review. Int J Environ Res Public Health. (2015) 12:4354–79. 10.3390/ijerph120404354 25913182 PMC4410252

[6] Deeg DJ Kriegsman DM. Concepts of self-rated health: specifying the gender difference in mortality risk. Gerontologist. (2003) 43:376–86. 10.1093/geront/43.3.376 12810902

[7] Pila E Sylvester BD Corson L Folkman C Huellemann KL Sabiston CM. Relative contributions of health behaviours versus social factors on perceived and objective weight status in Canadian adolescents. Can J Public Health. (2021) 112:464–72. 10.17269/s41997-020-00458-4 33428114 PMC8076362

[8] Wilson K Eyles J Ellaway A Macintyre S Macdonald L. Health status and health behaviours in neighbourhoods: a comparison of Glasgow, Scotland and Hamilton, Canada. Health Place. (2010) 16:331–8. 10.1016/j.healthplace.2009.11.001 20022285 PMC2954309

[9] Miao J Wu X. Urbanization, socioeconomic status and health disparity in China. Health Place. (2016) 42:87–95. 10.1016/j.healthplace.2016.09.008 27750075

[10] Yin L. Street level urban design qualities for walkability: combining 2D and 3D GIS measures. Comput Environ Urban Syst. (2017) 64:288–96. 10.1016/j.compenvurbsys.2017.04.001

[11] Maass R Kloeckner CA Lindstrøm B Lillefjell M. The impact of neighborhood social capital on life satisfaction and self-rated health: a possible pathway for health promotion? Health Place. (2016) 42:120–8. 10.1016/j.healthplace.2016.09.011 27770668

[12] Salvatore MA Grundy E. Area deprivation, perceived neighbourhood cohesion and mental health at older ages: a cross lagged analysis of UK longitudinal data. Health Place. (2021) 67:102470. 10.1016/j.healthplace.2020.102470 33212393

[13] Macmillan A Hosking J Connor J Bullen C Ameratunga S. A Cochrane systematic review of the effectiveness of organisational travel plans: improving the evidence base for transport decisions. Transp Policy. (2013) 29:249–56. 10.1016/j.tranpol.2012.06.019

[14] Kondo MC Fluehr JM McKeon T Branas CC. Urban green space and its impact on human health. Int J Environ Res Public Health. (2018) 15:445. 10.3390/ijerph15030445 29510520 PMC5876990

[15] Vaz C Andrade AC Silva U Rodríguez D Wang X Moore K. Physical disorders and poor self-rated health in adults living in four latin american cities: a multilevel approach. Int J Environ Res Public Health. (2020) 17:8956. 10.3390/ijerph17238956 33276424 PMC7730272

[16] Biao Z Qingxu L Gaodi X Yunting S. Pattern Change of Urban Green Space in Beijing from 2000 to 2010. J Landscape Res. (2017) 9:67–73. 10.16785/j.issn1943-989x2017.4.016

[17] Seto Karen C Fragkias Michail neralp G Burak Reilly Michael K. A meta-analysis of global urban land expansion. PLoS ONE. (2011) 6:e23777. 10.1371/journal.pone.0023777 21876770 PMC3158103

[18] Wang K Li Z Zhang J Wu X Jia M Wu L. Built-up land expansion and its impacts on optimizing green infrastructure networks in a resource-dependent city. Sustain Cities Soc. (2020) 55:102026. 10.1016/j.scs.2020.102026

[19] Sato Y Zenou Y. How urbanization affect employment and social interactions. Eur Econ Rev. (2015) 75:131–55. 10.1016/j.euroecorev.2015.01.011

[20] Akpinar A Barbosa-Leiker C Brooks KR. Does green space matter? Exploring relationships between green space type and health indicators. Urban For Urban Gree. (2016) 20:407–18. 10.1016/j.ufug.2016.10.013

[21] Markevych I Schoierer J Hartig T Chudnovsky A Hystad P Dzhambov AM. Exploring pathways linking greenspace to health: Theoretical and methodological guidance. Environ Res. (2017) 158:301–17. 10.1016/j.envres.2017.06.028 28672128

[22] Maas J Verheij RA de Vries S Spreeuwenberg P Schellevis FG Groenewegen PP. Morbidity is related to a green living environment. J Epidemiol Commun Health. (2009) 63:967–73. 10.1136/jech.2008.079038 19833605

[23] Kaplan S. The restorative benefits of nature: toward an integrative framework. J Environ Psychol. (1995) 15:169–82. 10.1016/0272-4944(95)90001-2

[24] Ulrich RS Simons RF Losito BD Fiorito E Miles MA Zelson M. Stress recovery during exposure to natural and urban environments. J Environ Psychol. (1991) 11:201–30. 10.1016/S0272-4944(05)80184-7

[25] Dzhambov AM Dimitrova DD. Urban green spaces' effectiveness as a psychological buffer for the negative health impact of noise pollution: a systematic review. Noise Health. (2014) 16:157. 10.4103/1463-1741.134916 24953881

[26] Jiang B Chang C-Y Sullivan WC. A dose of nature: Tree cover, stress reduction, and gender differences. Landsc Urban Plan. (2014) 132:26–36. 10.1016/j.landurbplan.2014.08.005

[27] Evenson KR Wen F Hillier A Cohen DA. Assessing the contribution of parks to physical activity using GPS and accelerometry. Med Sci Sports Exerc. (2013) 45:1981. 10.1249/MSS.0b013e318293330e 23531716 PMC3778115

[28] Giles-Corti B Broomhall MH Knuiman M Collins C Douglas K Ng K. Increasing walking: how important is distance to, attractiveness, and size of public open space? Am J Prev Med. (2005) 28:169–76. 10.1016/j.amepre.2004.10.018 15694525

[29] Dzhambov AM Browning MH Markevych I Hartig T Lercher P. Analytical approaches to testing pathways linking greenspace to health: A scoping review of the empirical literature. Environ Res. (2020) 186:109613. 10.1016/j.envres.2020.109613 32668553

[30] Fleming CM Manning M Ambrey CL. Crime, greenspace and life satisfaction: An evaluation of the New Zealand experience. Landsc Urban Plan. (2016) 149:1–10. 10.1016/j.landurbplan.2015.12.014

[31] Luo W Qi Y. An enhanced two-step floating catchment area (E2SFCA) method for measuring spatial accessibility to primary care physicians. Health Place. (2009) 15:1100–7. 10.1016/j.healthplace.2009.06.002 19576837

[32] Woo J Tang N Suen E Leung J Wong M. Green space, psychological restoration, and telomere length. Lancet. (2009) 373:299–300. 10.1016/S0140-6736(09)60094-5 19167568

[33] Ma J Dong G Chen Y Zhang W. Does satisfactory neighbourhood environment lead to a satisfying life? An investigation of the association between neighbourhood environment and life satisfaction in Beijing. Cities. (2018) 74:229–39. 10.1016/j.cities.2017.12.008

[34] Mears M Brindley P Jorgensen A Ersoy E Maheswaran R. Greenspace spatial characteristics and human health in an urban environment: an epidemiological study using landscape metrics in Sheffield, UK. Ecol Indic. (2019) 106:105646. English. 10.1016/j.ecolind.2019.105464

[35] Van Dillen SM de Vries S Groenewegen PP Spreeuwenberg P. Greenspace in urban neighbourhoods and residents' health: adding quality to quantity. J Epidemiol Community Health. (2012) 66:e8–e8. 10.1136/jech.2009.104695 21715445

[36] Taleai M Sliuzas R Flacke J. An integrated framework to evaluate the equity of urban public facilities using spatial multi-criteria analysis. Cities. (2014) 40:56–69. 10.1016/j.cities.2014.04.006

[37] Lättman K Olsson LE Friman M. Development and test of the Perceived Accessibility Scale (PAC) in public transport. J Transport Geography. (2016) 54:257–63. 10.1016/j.jtrangeo.2016.06.015

[38] McCormack GR Cerin E Leslie E Du Toit L Owen N. Objective versus perceived walking distances to destinations: correspondence and predictive validity. Environ Behav. (2008) 40:401–25. 10.1177/0013916507300560

[39] Park G Chung W. Self-rated health as a predictor of mortality according to cognitive impairment: findings from the Korean Longitudinal Study of Aging (2006-2016). Epidemiol Health. (2021) 43:e2021021. 10.4178/epih.e2021021 33831294 PMC8289473

[40] Bozick R. The utility of self-rated health in population surveys: the role of bodyweight. Popul Health Metr. (2021) 19:1–11. 10.1186/s12963-021-00255-2 33941193 PMC8091531

[41] Greenland S Mansournia MA Altman DG. Sparse data bias: a problem hiding in plain sight. BMJ. (2016) 352:i1981. 10.1136/bmj.i1981 27121591

[42] Gascon M Sánchez-Benavides G Dadvand P Martínez D Gramunt N Gotsens X. Long-term exposure to residential green and blue spaces and anxiety and depression in adults: a cross-sectional study. Environ Res. (2018) 162:231–9. 10.1016/j.envres.2018.01.012 29358115

[43] Wu L Kim SK. Health outcomes of urban green space in China: evidence from Beijing. Sustain Cities Soc. (2021) 65:102604. 10.1016/j.scs.2020.10260431835826

[44] Shanahan DF Bush R Gaston KJ Lin BB Dean J Barber E. Health benefits from nature experiences depend on dose. Sci Rep. (2016) 6:1–10. 10.1038/srep28551 27334040 PMC4917833

[45] Schipperijn J Cerin E Adams MA Reis R Smith G Cain K. Access to parks and physical activity: an eight country comparison. Urban For Urban Green. (2017) 27:253–63. 10.1016/j.ufug.2017.08.010 29805351 PMC5967254

[46] Yuan L Shin K Managi S. Subjective well-being and environmental quality: the impact of air pollution and green coverage in China. Ecol Econ. (2018) 153:124–38. 10.1016/j.ecolecon.2018.04.033

[47] Duan Y Wagner P Zhang R Wulff H Brehm W. Physical activity areas in urban parks and their use by the elderly from two cities in China and Germany. Landsc Urban Plan. (2018) 178:261–9. 10.1016/j.landurbplan.2018.06.009

[48] Kleerekoper L Van Esch M Salcedo TB. How to make a city climate-proof, addressing the urban heat island effect. Resour Conserv Recycl. (2012) 64:30–8. 10.1016/j.resconrec.2011.06.004

[49] Wang H Dai X Wu J Wu X Nie X. Influence of urban green open space on residents' physical activity in China. BMC Public Health. (2019) 19:1–12. 10.1186/s12889-019-7416-7 31409316 PMC6693084

[50] Bratman GN Daily GC Levy BJ Gross JJ. The benefits of nature experience: Improved affect and cognition. Landsc Urban Plan. (2015) 138:41–50. 10.1016/j.landurbplan.2015.02.005

[51] Zhang L Zhou S Kwan M-P. A comparative analysis of the impacts of objective versus subjective neighborhood environment on physical, mental, and social health. Health Place. (2019) 59:102170. 10.1016/j.healthplace.2019.102170 31422227

[52] Xie B An Z Zheng Y Li Z. Healthy aging with parks: association between park accessibility and the health status of older adults in urban China. Sustain Cities Soc. (2018) 43:476–86. 10.1016/j.scs.2018.09.010

